# Glycogen synthase kinase 3β promotes osteosarcoma invasion and migration via regulating PTEN and phosphorylation of focal adhesion kinase

**DOI:** 10.1042/BSR20193514

**Published:** 2021-07-26

**Authors:** Wei Mai, Lingyu Kong, Hongwei Yu, Junjie Bao, Chunyu Song, Guofan Qu

**Affiliations:** 1 Department of Orthopedics, Harbin Medical University Cancer Hospital, Harbin, Heilongjiang 150081, China; 2 Department of Head and Neck Surgery, Harbin Medical University Cancer Hospital, Harbin, Heilongjiang 150081, China

**Keywords:** Glycogen synthase kinase 3β, Human osteosarcoma, Metastasis

## Abstract

Aim: Typical features of human osteosarcoma are highly invasive and migratory capacities. Our study aimed to investigate the roles of glycogen synthase kinase 3β (GSK3β) in human osteosarcoma metastasis.

Methods: GSK3β expressions in clinical osteosarcoma tissues with or without metastasis were examined by immunohistochemical staining. The expressions of GSK3β, p-GSK3β^Ser9^, and p-GSK3β^Tyr216^ in human osteoblast cells (hFOB1.19) and human osteosarcoma cells (MG63, SaOS-2, and U2-OS) were detected by Western blotting. The GSK3β activity was measured by non-radio isotopic *in vitro* kinase assay. Migration and invasion abilities of MG-63 cells treated with small-molecular GSK3β inhibitors were respectively examined by monolayer-based wound-healing assay and transwell assay. The mRNA expressions of GSK3β, matrix metalloproteinase-2 (MMP-2), MMP-9, phosphatase with tensin homology (PTEN), and focal adhesion kinase (FAK) were detected after siRNA transfection for 72 h. Meanwhile, protein expressions of GSK3β, FAK, p-FAK^Y397^, PTEN, MMP-2, and MMP-9 were measured by Western blotting.

Results: Clinical osteosarcoma tissues with metastasis showed higher GSK3β expressions. MG63 and U2-OS cells that were easy to occur metastasis showed significantly higher expressions and activities of GSK3β than SaOS-2 cells. Inhibition of GSK3β with small-molecular GSK3β inhibitors in MG63 cells significantly attenuated cell migration and invasion. These effects were associated with reduced expressions of MMP-2 and MMP-9. Moreover, increased PTEN and decreased p-FAK^Y397^ expressions were observed following GSK3β knockdown by siRNA transfection. Conclusion: GSK3β might promote osteosarcoma invasion and migration via pathways associated with PTEN and phosphorylation of FAK.

## Introduction

Invasion and metastasis of human osteosarcoma often occur in the early stages, and the lungs are the most distant metastatic sites, accounting for about 90% of all metastases [1]. Until now, few reliable predictors have been identified to guide the choice or intensity of the human osteosarcoma therapy [2]. Though neoadjuvant chemotherapy could improve the survival rate of patients with osteosarcoma in last decades, invasive nature and pulmonary trend metastasis still remain to be the main reasons for the failure of osteosarcoma therapy [3]. Thus, revealing the signaling pathways involved in the metastatic process of human osteosarcoma has become an important aspect for developing novel effective therapeutic aimed at improving the survival rate of osteosarcoma [4].

Glycogen synthesis kinase 3β (GSK3β) plays an important role in cell growth, proliferation and cell migration, and it is widely concerned in the field of oncology. GSK3β is a serine/threonine protein kinase and has emerged as a key enzyme in regulating several important cellular signaling pathways via phosphorylating its substrates [5]. Nowadays, the developed small molecule inhibitors of GSK3β have been used to treat type II diabetes and Alzheimer disease in clinical [6–8]. Under normal physiological conditions, GSK3β negatively regulates cell survival and proliferation through phosphorylating multiple oncogene proteins (e.g., β-catenin) and carcinogenic transcription factors (e.g., c-Myc), which result in their ubiquitin degradation and inactivation. Thus, GSK3β is typically supposed as a ‘tumor-suppressor genes’ [9]. Our previous work demonstrated that the overexpression and abnormal activation of GSK3β in gastrointestinal cancer cells inhibit the apoptosis, and promote cell survival and proliferation [10]. In addition, reducing the activity or expression level of GSK3β inhibits the survival and proliferation of gastrointestinal cancer cell and induced cell apoptosis. Therefore, GSK3β has potentially to be a novel therapeutic target for gastrointestinal cancer [10,11].

In the study of Cai et al., GSK3β inhibitor treatment results in the decrease of cell survival and proliferation rate, indicating that GSK3β may be associated with the occurrence of osteosarcoma [12]. Tang et al. reported that overexpressing GSK3β in osteosarcoma cell significantly improved the colony formation and increased the tumor formation rate. Importantly, they demonstrated that the abnormal activation of GSK3β promoted the growth of osteosarcoma tumor [13]. Those findings revealed that GSK3β plays an important role in the tumorigenesis of osteosarcoma, but the underlying molecular mechanisms of GSK3β regulating the metastasis and invasion of osteosarcoma still remain unknown.

Here, we set out to explore the underlying molecular mechanisms of GSK3β in the metastasis of osteosarcoma via small molecular inhibitors and siRNA knock down analysis.

## Materials and methods

### Immunohistochemical staining of clinical samples

The collected osteosarcoma tissue specimens from patients with primary metastatic osteosarcoma (*n*=24) and patients with nonmetastatic osteosarcoma (*n*=16) were fixed in 10% formalin and embedded in paraffin. The lung metastasis was diagnosed by computed tomography (CT) imaging. Expression levels of GSK3β in primary osteosarcoma tissues with or without metastasis were examined by standard immunohistochemical staining [14]. Immunohistochemical staining was carried out by the avidin-biotin method using an ABComplex/HRP kit (Waitai, China). The tissue sections were deparaffinized, antigen retrieved by microwave and blocked of nonspecific immunoreactions. Next, tissue sections were incubated with primary antibodies against GSK3β (1:100, BD Biosciences). For the negative control, primary antibodies were replaced by nonimmune mouse IgG (Abcam). After rinsing in phosphate-buffered saline, tissue sections were then incubated with biotinylated secondary antibodies (1:2000; Abcam). Subsequently, the nuclei were counterstained with hematoxylin. The quantitative analysis of GSK3β expression levels in tumor samples of patients with primary metastatic osteosarcoma and patients with nonmetastatic osteosarcoma was performed by TissueFaxs software (TissueGnostics GmbH).

### Cell lines and cell culture

Human osteosarcoma cell lines (MG-63 and SaOS-2) and normal human osteoblast cell line (hFOB1.19) were purchased from the American Type Culture Collection (ATCC). Human osteosarcoma cells and osteoblast cells were cultured in DMEM and DMEM/Ham’s F12 mediums (Gibco, Thermo Fisher Scientific) respectively, supplemented with 10% fetal bovine serum. Another human osteosarcoma cell line U2-OS was obtained from the Human Cancer Cell Line Bank located in the Cancer Research Institute of Kanazawa University. U2-OS cells were cultured in RPMI-1640 (Sigma-Aldrich, St. Louis, MO) containing 10% fetal bovine serum. Cells were incubated at 37°C in a humidified atmosphere containing 5% CO_2_.

### Western blotting

The total cellular protein was extracted from cultured cells using lysis buffer (Sigma-Aldrich, St. Louis, MO) containing a mixture of protease and phosphatase inhibitors (Sigma-Aldrich). The total cellular protein samples were loaded on to sodium dodecyl sulfate (SDS) polyacrylamide gels and then transferred to nitrocellulose membranes (GE Healthcare Bio-Sciences, U.S.A.). After the membranes were blocked in blocking solution, the blots were incubated overnight at 4°C with primary antibodies. The primary antibodies were against GSK3β (1:1000; BD Biosciences), and its fractions phosphorylated at the serine^9^ residue (p-GSK3β^Ser9^; 1:1000; Cell Signaling Technology) and the tyrosine^216^ residue (p-GSK3β^Tyr216^; 1:1000; BD Biosciences); β-catenin (1:1000; BD Biosciences) and its fractions phosphorylated at S33, S37, and/or threonine 41 (T41) residues (p-β-catenin^S33/37/T41^); and β-actin (1:4000; Abcam); FAK (1:2000; Cell Signaling Technology); p-FAK^Y397^ (1:2000; Invitrogen); PTEN (1:2000; Cell Signaling Technology); MMP-2 and MMP-9 (1:3000; Abcam). Specific horseradish peroxidase (HRP)-conjugated secondary antibodies were used for following incubations overnight at 4°C. Immunoblotting signals were measured by the CS analyzer (ATTO, Japan).

### Nonradio isotopic *in vitro* kinase assay (NRIKA)

The nonradio isotopic *in vitro* kinase assay developed in our laboratory [10] was used to detect GSK3β activity in cultured cells. It uses a sequential combination of immunoprecipitations to isolate GSK3β in cellular protein extracted from cultured cells, an *in vitro* kinase reaction that uses recombinant β-catenin protein (substrate) and nonradio isotopic adenosine triphosphate, followed by immunoblotting to detect p-β-catenin^Ser33/37/Thr41^.

### Cell migration and invasion abilities

Migration and invasion abilities of MG-63 cells were respectively examined by monolayer-based wound-healing assay and transwell assay according the description of Ayako et al*.* [14]. Briefly, confluent monolayers of MG-63 cells in the presence of dimethyl sulfoxide (DMSO, control) or small-molecule GSK3β inhibitors (SB-216763, Sigma-Aldrich; AR-A014418, Calbiochem) at indicated concentrations (5, 10, 15, or 20 μmol/l) were scratched mechanically with a micropipette tip to create wound cell-free zone. The extent of cell migration was measured by the ability to close the artificially created gap. The gap distance between the wound edges was monitored at three fixed time points from 6 to 24 h by a CCD camera (Axiocam MRm, Zeiss) connected to a phase-contrast microscope (Axiovert 40 CFL, Zeiss).

MG-63 cell invasion ability was examined by the transwell assays using matrigel-coated 24-well double chamber system (BD BioCoat™ Matrigel™ Incubation Chamber, BD Bioscience). MG-63 cells were suspended in serum-free medium containing DMSO or small-molecule GSK3β inhibitors (SB-216763, AR-A014418; 20 μmol/l). Approximately 2 × 10^4^ cells were applied to the upper chamber pairing with the lower chamber suspended in medium containing 10% fetal bovine serum (as a chemo-attractant) and DMSO or small-molecule GSK3β inhibitors (SB-216763 or AR-A014418; 20 μmol/l). Cells on the lower side of the chamber were fixed and stained with Diff-Quick Kit (Symex) after incubation for 24 h. In each assay, the total number of cells per high-power microscopic field on the lower side of the matrigel coated chamber was counted and scored for invading cells. Meanwhile, the mRNA expressions of GSK-3β, MMP-2, and MMP-9 in MG63 cells under different treatments were measured by semiquantitative reverse transcription-PCR (RT-PCR). Glyceraldehide-3-phosphate dehydrogenase (GAPDH) was used as an internal control to monitor the efficiency of RT-PCR. The primers for GSK3β, MMP-2, and MMP-9 were designed and synthesized by Shanghai GenePharma.

### Small interfering RNA (siRNA) transfection

Small interfering RNA (siRNA) specific to human GSK3 (target sequence, 5′-GCUCCAGAUCAUGAGAAAGCUAGAU-3′; GSK3β Validated Stealth RNAi) and negative control siRNA (Stealth RNAi Negative Control Low GC duplex) were purchased from Invitrogen. MG63 cells were transfected with 20 nmol/l of either GSK3β-specific or negative control siRNA by using Lipofectamine RNAi MAX (Invitrogen) according to the manufacturer’s instructions. Effects of RNA interference on expression of GSK3β were determined by RT-PCR and Western blotting, respectively. Glyceraldehide-3-phosphate dehydrogenase (GAPDH) was used as an internal control to monitor the efficiency of RT-PCR. The primers for GSK3β, MMP-2, MMP-9, PTEN, and FAK for RT-PCR were designed and synthesized by Shanghai GenePharma (Shanghai, China). After siRNA transfection for 72 h, the protein expressions of GSK3β, FAK, p-FAK^Y397^, PTEN, MMP-2, and MMP-9 were analyzed by Western blotting as described above.

### Statistical analysis

Statistical analyses were performed using the SPSS software (version 16.0; SPSS Inc., Chicago, IL). All experiments were repeated at least three times to calculate the mean and standard deviation (SD). The Student’s *t-*test was conducted for normally distributed data. A *P*-value < 0.05 was regarded as statistically significant.

## Results

### Expressions of GSK3β in primary human metastatic or non-metastatic osteosarcoma tissues

**Figure 1:**
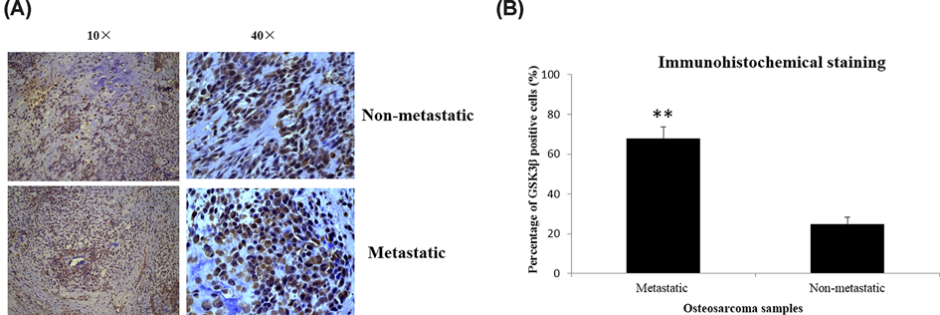
Measurements of GSK3β in tissue samples collected from patients with primary metastatic osteosarcoma and nonmetastatic osteosarcoma by immunohistochemical staining (**A**) Microscopy images of positive areas of GSK3β in tissue samples of non-metastatic and metastatic osteosarcoma. (**B**) Percentage of GSK3β positive cells in tissue samples of nonmetastatic and metastatic osteosarcoma. **P<0.01, compared with nonmetastatic osteosarcoma.

The expressions of GSK3β in tissue samples of 24 patients with primary metastatic osteosarcoma and 16 patients with nonmetastatic osteosarcoma were detected by the immunohistochemical staining. The immunohistochemistry staining results showed that the percentage of GSK3β positive cells in the tumor samples of patients with primary metastatic osteosarcoma was markedly higher than that of patients without metastasis (67.8 ± 5.8% vs. 24.8 ± 3.6%, *P*<0.001; Figure 1).

### Expressions, phosphorylation, and activity of GSK3β in human osteosarcoma and osteoblast cells

**Figure 2:**
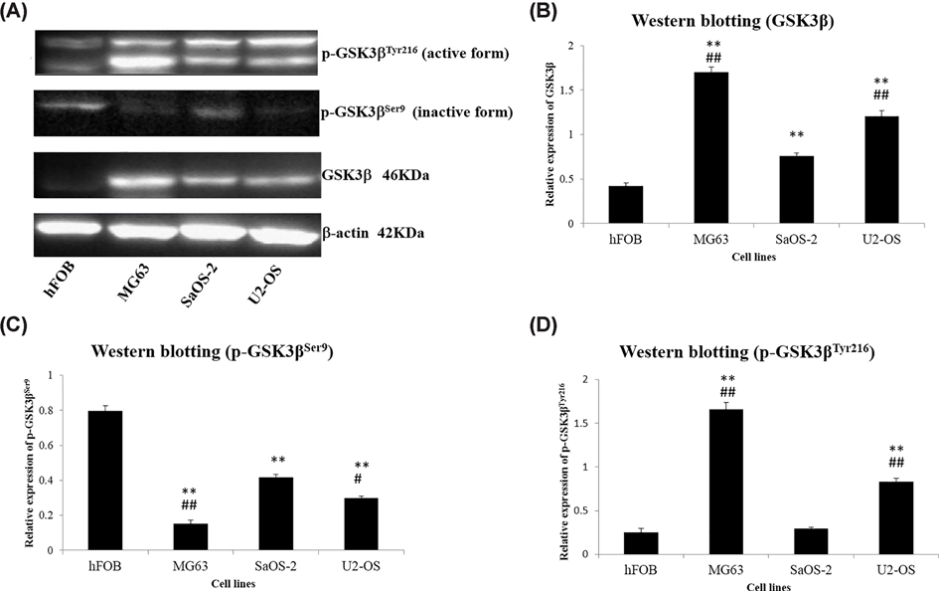
Protein expressions and phosphorylation of GSK3β in human osteosarcoma cells and normal human osteoblast cells (**A**) The GSK3β phosphorylated at the serine^9^ residue (p-GSK3β^Ser9^; inactive form) and the tyrosine^216^ residue (p-GSK3β^Tyr216^; active form) in protein extracts from three human osteosarcoma cells (MG63, SaOS-2, and U2-OS) and normal human osteoblast cells (hFOB1.19) were detected by Western blotting. (**B**) Relative expression of GSK3β in hFOB1.19, MG63, SaOS-2, and U2-OS cells. (**C**) Relative expression of p-GSK3β^Ser9^ in hFOB1.19, MG63, SaOS-2, and U2-OS cells. (**D**) Relative expression of p-GSK3β^Tyr216^ in hFOB1.19, MG63, SaOS-2, and U2-OS cells. **P<0.01, compared with hFOB1.19 cells; #*P*<0.05, ##*P*<0.01, compared with SaOS-2 cells.

Compared with normal human osteoblast cells (hFOB1.19), the human osteosarcoma cells (MG63, SaOS-2, and U2-OS) showed higher protein expression levels of GSK3β and active form of GSK3β (p-GSK3β^Tyr216^), while lower levels of inactive form of GSK3β (p-GSK3β^Ser9^) (Figure 2). Furthermore, the MG63 and U2-OS cells that were easy to occur metastasis showed significantly higher levels of GSK3β and active GSK3β (p-GSK3β^Tyr216^), and lower levels of inactive (p-GSK3β^Ser9^) compared with SaOS-2 cells (Figure 2).

**Figure 3:**
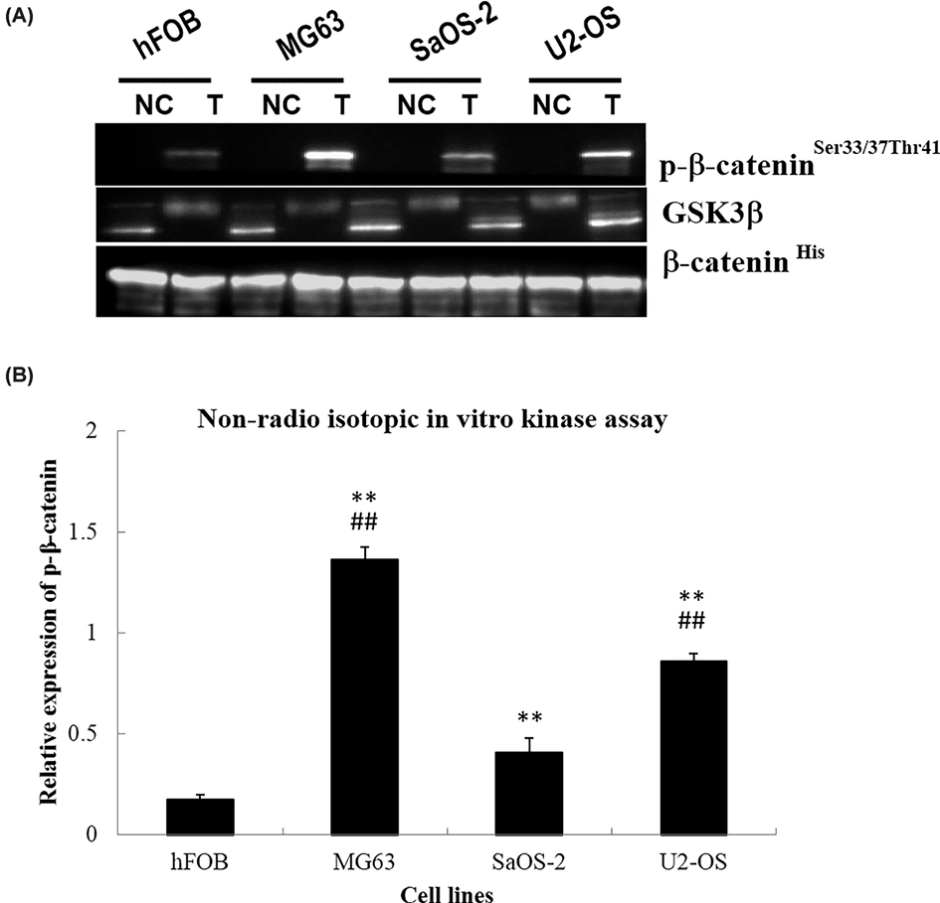
Detection of GSK3β activity by nonradio isotopic *in vitro* kinase assay (NRIKA) in three human osteosarcoma cells (MG63, SaOS-2, and U2-OS) and normal human osteoblast cells (hFOB1.19) (**A**) GSK3β activity to phosphorylate its substrate (β-catenin^His^) was demonstrated by expression of the phosphorylated β-catenin^His^ (p-β-catenin^Ser33/37/Thr41^) in the test lanes (T) and in the condition of little or no expression of p-β-catenin^Ser33/37/Thr41^ in the negative control reaction (NC). The amount of GSK3β and the presence of β-catenin^His^ (substrate) in the NRIKA reaction were monitored by immunoblotting with mouse monoclonal antibodies to GSK3β and β-catenin, respectively. Levels of β-catenin^His^ and its phosphorylated fraction (p-β-catenin^Ser33/37/Thr41^) in each reaction were measured by densitometric analysis of immunoblotting signals using a CS analyzer. (**B**) Relative expression of p-β-catenin in hFOB1.19, MG63, SaOS-2, and U2-OS cells. **P<0.01, compared with hFOB1.19 cells; ##*P*<0.01, compared with SaOS-2 cells.

In order to explore the activity of phosphorylating β-catenin, substrates of GSK3β, in human osteosarcoma cells and nontumor cells, we performed the NRIKA analysis in these cells. The human osteosarcoma cells exhibited stronger phosphorylating β-catenin activity compared with normal osteoblast cells hFOB1.19 (Figure 3). More specifically, the MG63 and U2-OS cells showed significantly higher activity of phosphorylating β-catenin when compared with SaOS-2 cells.

### Effects of GSK3β inhibitors on MG63 cell migration and invasion

**Figure 4:**
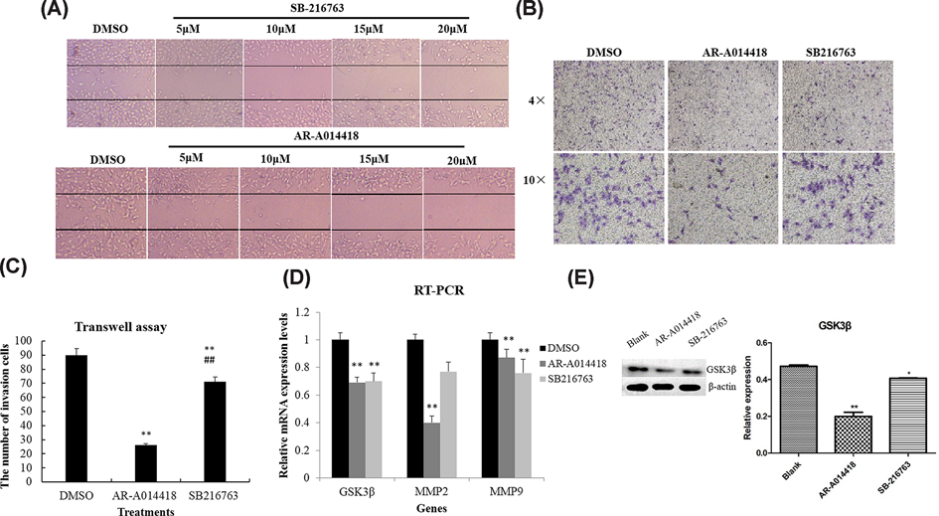
Effects of small molecule GSK3β inhibitors on the migration, invasion and related protein expressions of MG63 cells (**A**) Concentration effects on MG63 cells migration detected by wound-healing assay in the presence of DMSO, SB-216763 (5, 10, 1,5 or 10 μmol/l), or AR-A014418 (5, 10, 15, or 10 μmol/l). (**B**) Invading MG63 cells through matrigel-coating transwell after the treatments with DMSO, SB-216763 (20 μmol/l), or AR-A014418 (20 μmol/l). (**C**) Statistical analysis for invasion ability of MG63 under treatments with DMSO, SB-216763 (20 μmol/l), or AR-A014418 (20 μmol/l). (**D**) The mRNA expressions of GSK3β, MMP-2, and MMP-9 in MG63 cells treatments with DMSO, SB-216763 (20 μmol/l), or AR-A014418 (20 μmol/l). * *P*<0.05, compared with DMSO, ** P<0.01, compared with DMSO; # *P*<0.05, compared with AR-A014418, ##* P*<0.01, compared with AR-A014418.

To address the relationship of GSK3β with tumor metastasis, we tested the effects of small-molecular GSK3β inhibitors, AR-A014418 and SB-216763, on MG-63 cell migration and invasion. The wound healing assay showed that both of the two GSK3β inhibitors (AR-A014418 and SB-216763) significantly reduced the migration of MG-63 cells on a concentration depend manner compared with the DMSO treated cells (Figure 4A). In addition, AR-A014418 exhibited more effective inhibition on MG63 cell migration than SB-216763. The Transwell assay showed that both of the two GSK3β inhibitors significantly inhibited the invasion of MG63 cells (Figure 4B). The number of invasion cells when treated with AR-A014418 was significantly lower than treated with SB-216763 (Figure 4C).

The transcriptional activity of GSK3β inhibited by AR-A014418 and SB-216763 was also analyzed by RT-PCR. Both of the AR-A014418 and SB-216763 significantly inhibited the transcriptional activity of GSK3β. AR-A014418 exhibited more significant inhibition of GSK3β than SB-216763 (Figure 4D). Meanwhile, the AR-A014418 significantly inhibited the expressions of MMP-2 and MMP-9 (Figure 4D).

### Changes in the metastasis related mRNA and proteins following GSK-3β knockdown

**Figure 5:**
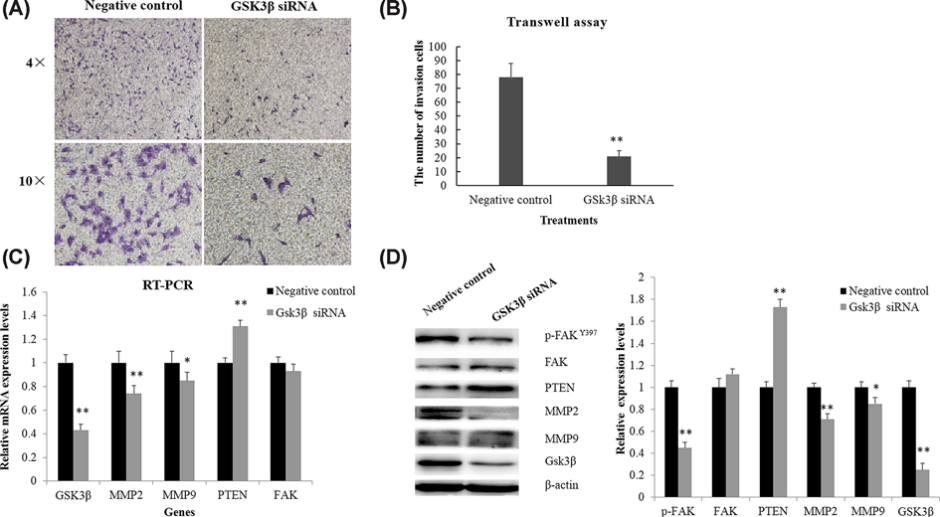
Changes in the invasion ability, gene, and protein expressions of MG63 cells following GSK3β knockdown by siRNA transfection for 72 h (**A**) Invading MG63 cells through matrigel-coating transwell after siRNA transfection. (**B**) Statistic analysis for invasion ability of MG63 cells under siRNA transfection. (**C**) The mRNA expressions of GSK3β, MMP-2, MMP-9, PTEN, and FAK in MG63 cells. (**D**) The protein expression of GSK3β, FAK, p-FAK^Y397^; PTEN, MMP-2, and MMP-9 in MG63 cells. *P<0.05, compared with negative control, **P<0.01, compared with negative control.

Moreover, GSK3β was knocked down by using RNA interference experiments. Consistent with the results of treatments with GSK3β inhibitors, GSK3β specific siRNA significantly reduced the invasion ability of MG63 cells (Figure 5A,B). The mRNA and protein expression levels of extracellular matrix enzyme MMP-2 and MMP-9 were also significantly reduced in MG-63 cells (Figure 5C,D). Meanwhile, the GSK3β knockdown significantly increased the expressions of PTEN in the mRNA level and protein level (Figure 5C,D). Though knocking down of GSK3β in MG63 cells did not significantly affect the total FAK expression, the protein expression of p-FAK^Y397^ was reduced (Figure 5C,D).

## Discussion

Although many studies have showed the pathogenic mechanism of osteosarcoma, few were focus on the underlying molecular mechanisms about metastasis of osteosarcoma [3]. Shimozaki et al. recently demonstrated a critical role for GSK-3β in sustaining survival and proliferation of osteosarcoma [15]. However, the roles of GSK-3β in the process of invasion and metastasis of osteosarcoma are still not clear. In our study, the expression of GSK-3β in osteosarcoma tissues from patients with metastasis is higher than that without metastasis. MG63 and U2-OS cells that were easy to occur metastasis showed significantly higher levels of GSK3β and active GSK3β (p-GSK3β^Tyr216^), and lower levels of inactive (p-GSK3β^Ser9^) compared with SaOS-2 cells. Furthermore, we found the activities of GSK-3β in MG63 and U2-OS cells were higher than that in SaOS-2 cells. These findings indicated that active GSK3β is correlated with human osteosarcoma metastasis.

In our study, inhibition of GSK-3β with small-molecular GSK3β inhibitors significantly reduced the migration and invasion ability of osteosarcoma cells. Meanwhile, the expressions of MMP-2 and MMP-9 were also reduced after treatments of small-molecular GSK3β inhibitors. MMP-2 and MMP-9 are highly expressed in osteosarcoma tissues, and they play important roles in the invasion and metastasis of tumor cells [16,17]. Thus, inhibition of GSK-3β may suppresses osteosarcoma cell migration and invasion by reducing the expressions of MMP-2 and MMP-9.

Moreover, the invasion ability of osteosarcoma cells following GSK3β knock-down by siRNA transfection for 72 h was obviously inhibited. The mRNA and protein expression levels of MMP-2 and MMP-9 were also significantly reduced. Phosphatase with tensin homology (PTEN) is a newly discovered tumor suppressor gene whose protein stability directly affects the migration of cells [18,19]. Furthermore, GSK-3β regulates the stability of PTEN via regulating the phosphorylation of PTEN, resulting in the ubiquitination and degradation of the PTEN [20]. The treatment of MG63 cells with GSK-3β siRNA transfection significantly increased the expression of PTEN in the mRNA level and protein level. The abnormal expression and phosphorylation levels of FAK in sarcoma are also reported to affect tumor cell migration and cell adhesion [21,22]. GSK3β regulates the phosphorylation of FAK, and GSK3β inhibitors reduce the phosphorylation level of the FAK [23,24]. Knocking down of GSK3β in MG63 cells did not significantly affect the total FAK in mRNA and protein, but reduced the p-FAK^Y397^, suggesting an involvement in the mechanism of GSK3β-mediated tumor metastasis. It has been reported that the secretions of MMP-2 and MMP-9 are negatively correlated with PTEN, while are positively correlated with FAK [25,26]. Therefore, it could be speculated that GSK-3β might promote the osteosarcoma metastasis by reducing the stability of PTEN and increasing the phosphorylation of FAK, and then accelerate the secretions of MMP-2 and MMP-9.

## Conclusion

In summary, our study revealed that inhibition of GSK-3β might suppress the osteosarcoma invasion and migration via the pathways associated with PTEN and phosphorylation of focal adhesion kinase, followed by reduced expression of MMP-2 or MMP-9.

## Data Availability

All data are provided within the paper.

## Abbreviations

ATCC: American Type Culture Collection
CT: computed tomography
FAK: focal adhesion kinase
GSK3β: glycogen synthase kinase 3β
MMP-2: matrix metalloproteinase-2
PTEN: phosphatase with tensin homology
SDS: sodium dodecyl sulfate
siRNA: small interfering RNA

## References

[1] Marulanda G.A., Henderson E.R., Johnson D.A., Letson G.D. and Cheong D. (2008) Orthopedic surgery options for the treatment of primary osteosarcoma. Cancer Control 15, 13–20 10.1177/107327480801500103 18094657

[2] Mirabello L., Troisi R.J. and Savage S.A. (2009) Osteosarcoma incidence and survival rates from 1973 to 2004 data from the surveillance, epidemiology, and end results program. Cancer 115, 1531–1543 10.1002/cncr.24121 19197972 PMC2813207

[3] Luetke A., Meyers P.A., Lewis I. and Juergens H. (2014) Osteosarcoma treatment - where do we stand? A state of the art review Cancer Treat. Rev. 40, 523–532 10.1016/j.ctrv.2013.11.006 24345772

[4] Khanna C., Fan T.M., Gorlick R., Helman L.J., Kleinerman E.S., Adamson P.C. et al. (2014) Toward a drug development path that targets metastatic progression in osteosarcoma. Clin. Cancer Res. 20, 4200–4209 10.1158/1078-0432.CCR-13-2574 24803583 PMC4134738

[5] Doble B.W. and Woodgett J.R. (2003) GSK-3: tricks of the trade for a multi-tasking kinase. J. Cell Sci. 116, 1175–1186 10.1242/jcs.00384 12615961 PMC3006448

[6] Jee S.H., Ohrr H., Sull J.W., Yun J.E., Ji M. and Samet J.M. (2005) Fasting serum glucose level and cancer risk in Korean men and women. Jama-J. Am. Med. Assoc. 293, 194–202 10.1001/jama.293.2.194 15644546

[7] Cohen P. and Goedert M. (2004) GSK3 inhibitors: development and therapeutic potential. Nat. Rev. Drug Discov. 3, 479–487 10.1038/nrd1415 15173837

[8] King M.R., Anderson N.J., Guernsey L.S. and Jolivalt C.G. (2013) Glycogen synthase kinase-3 inhibition prevents learning deficits in diabetic mice. J. Neurosci. Res. 91, 506–514 10.1002/jnr.23192 23362012 PMC3570718

[9] Manoukian A.S. and Woodgett J.R. (2002) Role of glycogen synthase kinase-3 in cancer: regulation by Wnts and other signaling pathways. Adv. Cancer. Res. 84, 203–229 10.1016/S0065-230X(02)84007-6 11883528

[10] Mai W., Kawakami K., Shakoori A., Kyo S., Miyashita K., Yokoi K. et al. (2009) Deregulated GSK3 beta sustains gastrointestinal cancer cells survival by modulating human telomerase reverse transcriptase and telomerase. Clin. Cancer Res. 15, 6810–6819 10.1158/1078-0432.CCR-09-0973 19903789

[11] Shakoori A., Ougolkov A., Yu Z.W., Zhang B., Modarressi M.H., Billadeau D.D. et al. (2005) Deregulated GSK3 beta activity in colorectal cancer: Its association with tumor cell survival and proliferation. Biochem Bioph. Res. Co. 334, 1365–1373 10.1016/j.bbrc.2005.07.041 16043125

[12] Cai Y.P., Mohseny A.B., Karperien M., Hogendoom P.C.W., Zhou G.Y. and Cleton-Jansen A.M. (2010) Inactive Wnt/beta-catenin pathway in conventional high-grade osteosarcoma. J. Pathol. 220, 24–33 10.1002/path.2628 19882675

[13] Tang Q.L., Xie X.B., Wang J., Chen Q., Han A.J., Zou C.Y. et al. (2012) Glycogen synthase kinase-3 beta, NF-kappa B signaling, and tumorigenesis of human osteosarcoma. J. Natl. Cancer I. 104, 749–763 10.1093/jnci/djs210 PMC3352834 22534782

[14] Kitano A., Shimasaki T., Chikano Y., Nakada M., Hirose M., Higashi T. et al. (2013) Aberrant glycogen synthase kinase 3β is involved in pancreatic cancer cell invasion and resistance to therapy. PLoS ONE 8, e55289 10.1371/journal.pone.0055289 23408967 PMC3568118

[15] Shimozaki S., Yamamoto N., Domoto T., Nishida H., Hayashi K., Kimura H. et al. (2016) Efficacy of glycogen synthase kinase-3beta targeting against osteosarcoma via activation of beta-catenin. Oncotarget 7, 77038–77051 10.18632/oncotarget.12781 27780915 PMC5363568

[16] Hyun-Ji C., Tae-Sung L., Jae-Bok P., Kwan-Kyu P., Jung-Yoon C., Doo-Il S. et al. (2007) Disulfiram suppresses invasive ability of osteosarcoma cells via the inhibition of MMP-2 and MMP-9 expression. J. Biochem. Mol. Biol. 40, 1069–1076 18047805 10.5483/bmbrep.2007.40.6.1069

[17] Jian Z., Xiaobing Z., Hengyuan L., Binghao L., Lingling S., Tao X. et al. (2015) Piperine inhibits proliferation of human osteosarcoma cells via G2/M phase arrest and metastasis by suppressing MMP-2/-9 expression. Int. Immunopharmacol. 24, 50–58 10.1016/j.intimp.2014.11.012 25479727

[18] Langlois M.J., Bergeron S., Bernatchez G., Boudreau F., Saucier C., Perreault N. et al. (2010) The PTEN Phosphatase Controls Intestinal Epithelial Cell Polarity and Barrier Function: Role in Colorectal Cancer Progression. PLoS ONE 5, 10.1371/journal.pone.0015742 PMC3009737 21203412

[19] Saini M.K. and Sanyal S.N. (2012) PTEN regulates apoptotic cell death through PI3-K/Akt/GSK3 beta signaling pathway in DMH induced early colon carcinogenesis in rat. Exp. Mol. Pathol. 93, 135–146 10.1016/j.yexmp.2012.04.019 22561258

[20] Maccario H., Pereira N.M., Davidson L., Downes C.P. and Leslie N.R. (2007) PTEN is destabilized by phosphorylation on Thr(366). Biochem. J. 405, 439–444 10.1042/BJ20061837 17444818 PMC2267318

[21] Perry B.C., Wang S.Y. and Basson M.D. (2010) Extracellular pressure stimulates adhesion of sarcoma cells via activation of focal adhesion kinase and Akt. Am. J. Surg. 200, 610–614 10.1016/j.amjsurg.2010.07.013 21056138 PMC3837573

[22] Yui Y., Itoh K., Yoshioka K., Naka N., Watanabe M., Hiraumi Y. et al. (2010) Mesenchymal mode of migration participates in pulmonary metastasis of mouse osteosarcoma LM8. Clin. Exp. Metastas 27, 619–630 10.1007/s10585-010-9352-x 20872237

[23] Bianchi M., De Lucchini S., Marin O., Turner D.L., Hanks S.K. and Villa-Moruzzi E. (2005) Regulation of FAK Ser-722 phosphorylation and kinase activity by GSK3 and PP1 during cell spreading and migration. Biochem. J. 391, 359–370 10.1042/BJ20050282 15975092 PMC1276935

[24] John J.K., Paraiso K.H.T., Rebecca V.W., Cantini L.P., Abel E.V., Pagano N. et al. (2012) GSK3 beta inhibition blocks melanoma cell/host interactions by downregulating N-cadherin expression and decreasing FAK phosphorylation. J. Invest. Dermatol. 132, 2818–2827 10.1038/jid.2012.237 22810307 PMC3479306

[25] Comincini S., Paolillo M., Barbieri G., Palumbo S., Sbalchiero E., Azzalin A. et al. (2009) Gene expression analysis of an EGFR indirectly related pathway identified PTEN and MMP9 as reliable diagnostic markers for human glial tumor specimens. J. Biomed. Biotechnol. 10.1155/2009/924565 19657395 PMC2718324

[26] Chen J.Y., Tang Y.A., Huang S.M., Juan H.F., Wu L.W., Sun Y.C. et al. (2011) A Novel Sialyltransferase Inhibitor Suppresses FAK/Paxillin Signaling and Cancer Angiogenesis and Metastasis Pathways. Cancer Res. 71, 473–483 10.1158/0008-5472.CAN-10-1303 21224350

